# Evaluation of Culture Conditions to Obtain Fatty Acids from Saline Microalgae Species:* Dunaliella salina*,* Sinecosyfis *sp., and* Chroomonas *sp.

**DOI:** 10.1155/2016/5081653

**Published:** 2016-06-08

**Authors:** D. A. Castilla Casadiego, A. R. Albis Arrieta, E. R. Angulo Mercado, S. J. Cervera Cahuana, K. S. Baquero Noriega, A. F. Suárez Escobar, E. D. Morales Avendaño

**Affiliations:** ^1^Engineering Faculty, Chemical Engineering Program, Bioprocess Research Group, Universidad del Atlántico, Km 7 Antigua Vía a Puerto Colombia, Barranquilla, Colombia; ^2^Basic Sciences Faculty, Chemistry Program, Biotechnology of Microalgae Research Group, Universidad del Atlántico, Km 7 Antigua Vía a Puerto Colombia, Barranquilla, Colombia; ^3^Engineering Faculty, Universidad de Bogotá Jorge Tadeo Lozano, 4 Avenue No. 22-61, Bogotá, Colombia; ^4^Sciences Experimental Faculty, Universidad del Zulia, Calle 61 con Prolongación Avenida 77, Maracaibo 4011, Venezuela

## Abstract

The use of the saline microalgae,* Dunaliella salina, Sinecosyfis *sp., and* Chroomonas *sp., was explored as an alternative source for the production of fatty acids using fertilizer and glycerol as culture media. The nutrient medium used contained “Nutrifoliar,” a commercial fertilizer, and/or glycerol, in natural sea water. The microalgae were placed in cultures with different conditions. The parameters that favored the largest production of fatty acids were 24 hours of agitation and illumination, 1620 L/day of air supply, 2.25 L of air/min, and a temperature of 32°C using “Nutrifoliar” as the culture media. Results indicated that, from 3 g of microalgae in wet base of* Chroomonas* sp., 54.43 mg of oil was produced. The chromatographic characterization of oil obtained revealed the presence of essential fatty acids such as 9,12,15-octadecatrienoic acid (omega-3) and 4,7,10-hexadecatrienoic acid (omega-6) from the species* Dunaliella salina. *On the other hand, 9,12-octadecadienoic acid (omega-6) and cis-11-eicosenoic acid (omega-9) were identified from the species* Chroomonas *sp. The temperature variations played an important role in the velocity of growth or the production of the algae biomass, the amount of oil, and the ability to produce fatty acids.

## 1. Introduction

Essential fatty acids are divided into two main families: omega-3 (n-3) and omega-6 (n-6), which are denominated as polyunsaturated fatty acids (PUFAs). This is due to the fact that its carbon chains are not saturated with hydrogen atoms and have more than one double bond between carbon atoms [1]. There are certain fatty acids that our organism is not able to synthesize, called essential fatty acids. These are of vital importance, contributing to the health of human beings and therefore must be administered through a person's diet [2].

A normal consumption of PUFAs has been associated with good health of human beings [3]. Fatty acids play a role in the development and maintenance of correct cerebral function, especially in the gestation period and childhood [4]. Several researches show that PUFAs protect and help reduce risk of contracting cardiovascular illnesses [5], diseases in premature babies [6], in the lactation and pregnancy period [7], ocular diseases associated with age [8], Alzheimer's disease [9], inflammatory illnesses [10], diabetes prevention [11], and certain types of cancer [12]. Finally, these lipids can be used for treatment of mental illnesses [13] and weight loss [14].

Currently, omega-3 fatty acids are obtained from sources such as fish oil, salmon, tuna, halibut, anchovy or herring, shrimp, and dried fruit oils [15]. Also, omega-6 fatty acids can be found in foods such as vegetable oil like soy, safflower, or corn; in dried fruit and seeds [16]; and in small amounts in meat, birds, and eggs [17].

One of the biggest problems to obtain these fatty acids from fish oil is their residual taste, which is not pleasant for the human palate, reducing its consumption and market sales. Due to the refinement process of this oil, necessary to eliminate the residual taste, the final product is more expensive, and on account of this alternative ways to obtain these lipids have been proposed in order to replace PUFAs from fish oil, using as feedstock another type of marine life like microalgae [15].

Recently, it has been proved that certain species of microalgae accumulate, under specific culture conditions, a good amount of fatty acids [15]. In particular, some saline strains present high percentages of omega oils that allow the exploration of obtaining, on a commercial scale, essential fatty acids such as docosahexaenoic acid (DHA) and eicosapentaenoic acid (EPA). Various studies developed with different microalgae used to obtain these fatty acids are shown in Table 1.

With the purpose of understanding which other species of saline algae can be good precursors to obtain fatty acids, this study presents as the principal objective to determine the potential of the species of saline microalgae,* Dunaliella salina, Sinecosyfis *sp., and* Chroomonas *sp., as sources of essential fatty acids, evaluating the best culture conditions that could help generate a larger production of algae biomass, amount of oil, and the ability to produce fatty acids of interest. Besides, this study explores the alternative of using commercially available feedstock, such as commercial fertilizers and glycerol, for the formulation of the growth medium of the culture of saline microalgae in order to reduce the cost associated with nitrogen and carbon sources for the production of fatty acids omega-3 using microalgae.

## 2. Materials and Methods

### 2.1. Microorganisms

The microalgae used correspond to three species:* Dunaliella salina, Chroomonas *sp., and* Sinecosyfis* sp. These species were donated by the Ceparium of Photosynthetic Microorganism's Laboratory of the Biology, Department of the Experimental Science Faculty, the Universidad del Zulia, Maracaibo, Venezuela. These strains were provided in test tubes with 10 mL of algal culture medium containing 4 mM of nitrogen and a salinity of 3.5%.

### 2.2. Nutrients and Culture Conditions

The algae were submerged in different conditions of growth, alternating the nutrient medium to obtain biomass: commercial “Nutrifoliar” fertilizer with a 4 mM concentration of nitrogen and glycerol at 4 mM of carbon, both in filtrated sea water and autoclaved. The cultures were at controlled temperatures of 20°C and 32°C. The artificial illumination was supplied through fluorescent lamps (Sylvania Daylight plus F96T12/DLP) of 75 W for all the cultures, with a luminous flow of 16880 lm, (16 lamps). The illumination source was regulated: a continuous exposition was supplied for selected cultures and others were submerged at a photoperiod of light : darkness 12 h : 12 h. The agitation and supply of air were provided through air pumps (PowerLife P -500), which provide 2.25 L of air/min with the purpose of avoiding the sedimentation of algae and permit culture homogenization.

Natural sea water was obtained from Salgar Beach in the Atlantic Coast, Colombia, coordinate latitude 11°1′16.62′′N, longitude 74°56′7.24′′W. The salinity of the sterile sea water was measured with conductivity electrodes, WTW brand and Multi 3420 model. The registered value was 37.5% and 56.2 mS/cm, for salinity and conductivity, respectively. All the materials used were previously sterilized in autoclave at 121°C.

### 2.3. Cell Growth

Cell growth was determined by taking daily samples of 5 mL from each of the different cultures and determining the concentration of microalgae spectroscopically (Spectronic Genesys 2), measuring the absorbance at a wavelength of 647 nm and monitoring each culture in fixed 24-hour intervals. Moisture content in centrifuged microalgae was obtained, drying samples of microalgae at 105°C until constant weight.

### 2.4. Lipid Extraction

The lipid fraction of microalgae was extracted using Soxhlet method, which is based on the constant evaporation and condensation through stages of a volatile solvent for posterior contact with the sample studied. Quantities of the biomass between 1.5 and 2.4 grams were weighed for each microalga and were subjected to Soxhlet extraction using analytic hexane (JT Baker) as solvent.

A mixture of hexane and lipids was obtained from the process of extraction. This mixture was separated through a rotary evaporator Büchi Heating Bath Model B-490 at 48°C, with a rotation velocity 64–66 rpm, for 45 minutes.

### 2.5. Determination of the Fatty Acids Profile

The extracted oils were transesterified, adding 1 mL of a methanolic solution of NaOH 1%. The mixture was heated to 55°C for 15 min and then 2 mL of a methanol solution containing 2% of HCl was added and heated for another 15 min [22]. Later, the obtained FAMEs were dissolved in 1 mL of hexane and injected to an Agilent 7890 A gas chromatograph, coupled to a selective mass detector 5975C (Agilent), with an Agilent Select Biodiesel for FAME column (30 m, 0.20 mm, 0.25 *μ*m), using Helium as a carrier gas with a flow of 1 mL/min and a volume of injection of 1 *μ*L, with a temperature programmed at 60°C for 2 minutes and then incremented in 15°C/min until a final temperature of 240°C was reached, with a time duration of 10 minutes. The injector and detector temperature were of 250°C according to EN 14103. The total time of analysis was 36.3 minutes.

### 2.6. Experimental Design

The experimental conditions evaluated are presented in Table 2.

Six experiments were established to develop this investigation and these are presented in Table 3, alternating the levels (− or +) which symbolize the different conditions or factors of experimentation presented in Table 2. Experiments were done in triplicate.

## 3. Results and Discussion

Some of the conditions studied according to the experimental design were no favorable for the algae growth. In Table 4, the conditions where the algae showed an increase of population are marked with “YES,” and those where the algae was not able to adapt are marked with “NO.”

Table 4 allows concluding that the microalgae have a higher affinity for the media formulation composed by the commercial fertilizer, “Nutrifoliar,” when compared to the culture media enriched with glycerol. Clearly, this can be observed for experimental conditions 4 and 6 reported in Table 4, where it is demonstrated that none of the microalgae species were able to survive in the media containing glycerol under these experimental conditions, but an acceptance of glycerol was seen when the experimental conditions 5 showed Table 3 were employed, which refers to the conditions of agitation, illumination, and air supply in low levels. The strains,* Dunaliella salina *and* Chroomonas *sp., were able to show a pattern of effective growth. The opposite effect was presented under the fourth-experimental conditions with high levels of agitation time, illumination, and air supply, which led to the death of this microalga. The species,* Sinecosyfis *sp., did not grow at any of the experimental conditions with glycerol. These can be explained either by the rapid depletion of the initial nitrogen contained in sea water when no additional nitrogen is added as it was done in the experiment that uses the culture media enriched with glycerol, or by osmotic stress due to the presence of glycerol in the enriched media, or by the combination of both phenomena.

Evaluating the effect of the variable temperature, experiments 1 and 3 reported that microalgae can grow under established conditions of 20 and 32°C. On the other hand, the species* Chroomonas *sp. was able to live through the established conditions of low levels of agitation time, illumination, and air supply, which make reference to experiment 2, Table 4.

**Figure 1:**
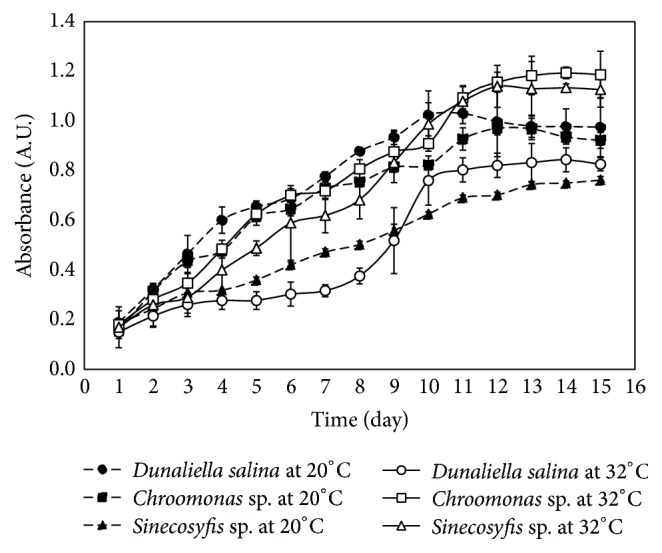
Comparative growth curves of* Dunaliella salina*,* Sinecosyfis *sp.,* and Chroomona *sp. microalgae, employing the commercial fertilizer, in conditions 20 and 32°C of temperature, agitation and illumination of 24 hours, and air supply of 1620 L/day.

**Figure 2:**
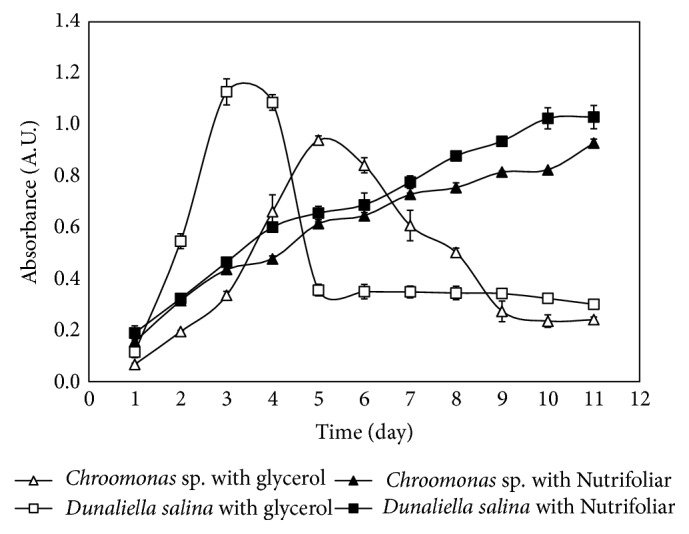
Comparative growth curves that represent absorbance versus time in days of microalgae* Dunaliella salina and Chroomonas *sp., employing glycerol and “Nutrifoliar” as nutrient mediums, at 20°C, an agitation and illumination of 24 hours, and an air supply of 1620 L/day.

Figures 1 and 2 show kinetics of growth of the microalgae species that were able to adapt and grow, under different conditions established in Table 4.

Figure 1 shows that the temperature at which the microalgae culture grew is an influential and selective parameter for the microalgae species, exerting an effect in the concentration of biomass in time. The microalgae culture of* Sinecosyfis* sp. and* Chroomonas *sp. at 32°C showed the highest concentrations of biomass, generating a reason for accumulation each day of 1.53 and 1.23 times more for the* Chroomonas* sp. and* Sinecosyfis* sp. microalgae, respectively, in the 11 days of culture, in comparison with the same strains of microalgae exposed to a growth below 20°C. The growth of* Dunaliella salina *microalgae under these two conditions of temperature demonstrated that this microalga is selective at temperatures around 20°C, presenting a higher concentration of almost double the biomass, in comparison with cultures exposed at 32°C. Other researchers that had studied the* Chlorella *sp.,* Nannochloropsis *sp. [23], and* Isochrysis galbana* microalgae [24] have reported that cultures exposed to moderately warm temperatures and high levels of light are able to grow significantly more. This is due to the increment in temperature, which favorably contributes to the photosynthesis [25] and respiration [26], reflecting an accelerated algal growth [23, 27].  Table 5 describes the behavior or stages of kinetic growth of microalgae seen in Figure 1. These cultures were recollected once they achieved the stationary growing phase to proceed with the oil extraction. One control for each culture was allowed to continue its growing, showing the final death phase for every culture. After centrifugation, wet microalgae showed average moisture of 68.8%.

Figure 2 shows that microalgae have a favorable acceptance of “Nutrifoliar” as its nutrient medium. This is due to the fact that the adaptation phase of the culture was less relevant in the kinetics described in Table 5, going straight to the linear phase, followed by the exponential phase (in some cases), stationary phase, and death phase. The highest absorbance data was achieved at 32°C, with 1.187 absorbance units with* Chroomonas* sp. microalgae and 1.127 absorbance units with* Sinecosyfis* sp. Both results were reported after 15 days of culture growth. At 32°C, the microalgae* Dunaliella salina *demonstrated little acceptance, due to the fact that it presented an adaptation phase for more than seven days, starting with an absorbance of 0.17 at the first day to 0.317 after seven days at 32°C. Evaluating its growth at 20°C, its concentration went from 0.18 of absorbance the first day to 0.77 after the seven days of culture. At 20°C, the exponential phase was not projected for* Sinecosyfis* sp. and* Chroomonas* sp. microalgae, causing it not to be able to grow with vitality and firmness; for this reason the lowest biomass concentration was generated.

Figure 2 shows glycerol as a promising nutrient medium to achieve high concentrations of biomass in a short period of time. Observing Figure 2 in detail, the amount of biomass obtained in three days using this nutrient source was able to surpass the amount obtained using a Nutrifoliar culture in 11 days, for* Dunaliella salina *cultures, with a ratio of biomass concentration of about 3 times more per day. The* Chroomonas* sp. microalgae cultures, nourished with glycerol, also showed the same behavior observed in the* Dunaliella salina *cultures: the amount of biomass that was accumulated using “Nutrifoliar” in 11 days was obtained in only 5 days in a glycerol culture, at a growth ratio of about twice as much.

It could also be appreciated in the useful culture lifetime. The strains were only able to bear 4 to 5 days for the cultures nourished with glycerol, in comparison with “Nutrifoliar,” which kept them alive for 11 days. The beginning of the cellular death phase occurred at an early stage for cultures with glycerol. This suggests that the microalgae studied, due to its mixotrophic character, can use this substance as a source of carbon for growth, causing a vertiginous growth during the exponential phase, with the absence of the adaptation stage. However, this rapid growth also caused an early disappearance of other nutrients in the culture medium (possibly nitrogen), and therefore its unviability, generating a rapid decay in biomass concentration. Gallardo and Cobelas [28] presented that one of the factors for the massive production of algae is the source of carbon and nitrogen, due to the fact that the absence of these would generate disequilibrium in the cultures. For this reason, even though it generated great density of microalgae biomass, the reported results of this work with the use of glycerol (carbon source) as a culture medium did not have the capacity necessary to maintain the culture without invigorated nitrogen.

Table 6 presents the results of the oil characterization produced by each microalgae biomass using different culture conditions, revealing the fatty acids that were identified in the samples.

Table 6 shows the results of the chromatographic characterization of the experiments that succeeded in the production of oil. These correspond to experimental conditions 1, 2, and 3 of Table 3, in which the nutrient medium employed was “Nutrifoliar.” Those cultures that reported growth and generation of high levels of biomass, with glycerol as a nutrient source, did not provide a significant amount of oil after the extraction process. This led to the conclusion that the series of culture conditions expressed in experiment 5 of Table 3, even though a high level of biomass was produced in a short period of time, were not able to stress the microalgae to concentrate oil in its interior.

From the experimental conditions 1 (Table 3), only the results for* Chroomonas* sp. and* Dunaliella salina *microalgae are shown.* Sinecosyfis* sp. did not produce a significant amount of oil. From the experimental conditions 2, only the results for* Chroomonas *sp. are showed. For the conditions studied in experiment 3,* Chroomonas *sp. and* Sinecosyfis *sp., cultures that were able to survive, did not guarantee a considerable production of oil.

Temperature was a crucial variable that favored the production of oil and the extraction of fatty acids, because, by submitting the cultures at 20°C, the amount of oil obtained and the extraction of fatty acids decreased in comparison with the conditioning of the cultures at 32°C. Our results reported that, for each gram of wet microalgae biomass of* Chroomonas* sp. at 32°C (experimental conditions 1), 18.14 mg of oil was produced and 5 different fatty acids were extracted, while, for each gram of biomass of* Chroomonas* sp. at 20°C (experimental condition 3), 2.62 mg of oil was obtained and only 3 types fatty acids were extracted. For the experimental conditions 2, where only the agitation and illumination period were fixed at 12 : 12 h, supplied with 810 L/day of air and at 32°C, the generation of oil was 16.66 mg for each gram of wet microalgae, obtaining a very small variation in comparison with results obtained in experiment 1, stressing the fact that the temperature has more influence in the production of oil than the other variables. The production of oil of the* Sinecosyfis *sp. microalga was also affected by temperature, reporting that, for each gram of biomass, 3.69 mg of oil was obtained. This is due to the fact that the increment in temperature was able to stress the microalgae, favoring a greater generation in the amount of oil.

Within the culture conditions evaluated, the one that favored the production of essential fatty acids with microalgae was 24 hours of agitation and 1620 L/day of air supply, 32°C, employing Nutrifoliar at 4 mM of nitrogen as the culture medium (experimental conditions 1). At these conditions, microalgae* Chroomonas* sp. and* Dunaliella salina* produced 4 and 5 different fatty acids (Table 6), respectively, among them 9,12,15-octadecatrienoic acid (omega-3) and 4,7,10-hexadecatrienoic acid (omega-6) for* Dunaliella salina*; these fatty acids were also identified in the research developed by Bhosale and collaborators in 2010 [19] with this same microalgae and 9,12-octadecadienoic acid (omega-6) with* Chroomonas* sp. microalgae. Also, hexadecanoic acid and heptadecanoic acid were the most common fatty acids, reported in all the analysis for the samples of different microalgae, at diverse culture conditions.

## 4. Conclusions

The nutrient source is the main variable that affected the growth and biomass production of the microalgae. The results showed that the glycerol, which only provides the carbon source to the cultures, was useful to produce around of the same microalgae biomass concentration that “Nutrifoliar” gained in 11 days in only 3 days with the microalgae* Dunaliella salina* and 5 days to* Chroomonas* sp. Low yields of oil production were obtained using glycerol as nutrient source; therefore, the extraction of fatty acids was not successful. “Nutrifoliar” was the nutrient source that all the studied microalgae accepted the most, with almost all the different experimental conditions being favorable for the cultures. The temperature was the most influential variable, generating significant contributions on the microalgae biomass and oil production and for obtaining fatty acids. At 32°C, the microalgae,* Sinecosyfis* sp. and* Chroomonas* sp., were able to grow and produced oil. At 20°C, the microalgae* Dunaliella salina *grew but did not produce oil in a quantifiable amount. The parameters that favored the largest production of oil and extraction of essential fatty acids were 24 hours of agitation and illumination, 1620 L/day of air supply and 2.25 L of air/min, and a temperature of 32°C using Nutrifoliar as culture medium. At these conditions, the* Chroomonas* sp. and* Dunaliella salina *produced polyunsaturated fatty acids of the omega-3 and omega-6 families.

## Figures and Tables

**Table 1:** Values (%) of EPA and DHA fatty acid contents with relations to total lipids in some microalgae [16].

Microalgae	% of EPA and DHA production	Reference
*Nannochloropsis salina*	~28 EPA	[17]
*Thraustochytrium *sp.	45.1 EPA + DHA	[18]
*Dunaliella salina*	21.4 EPA	[19]
*Pavlova viridis*	36.0 EPA + DHA	[20]
*Isochrysis galbana*	~28.0 EPA + DHA	[21]

**Table 2:** Variables and factors of experimentation.

Factors (*F*)	Variables	Levels
*F*1: nutrient type	Nutrifoliar	−
	Glycerol	+
*F*2: temperature (°C)	20	−
	32	+
*F*3: agitation time (h)	12	−
	24	+
*F*4: photoperiod light : darkness (h)	12 : 12	−
	24 : 0	+
*F*5: air supply (L/day)	810	−
	1620	+

**Table 3:** Experimental factors level combinations.

Experimental condition	*F*1	*F*2	*F*3	*F*4	*F*5
1	−	+	+	+	+
2	−	+	−	−	−
3	−	−	+	+	+
4	+	+	+	+	+
5	+	+	−	−	−
6	+	−	+	+	+

**Table 4:** Results of culture condition evaluations and treatment of the microalgae species subject to studies.

Experimental condition	Microalgae/evaluation of culture conditions and treatment		
	*Dunaliella salina*	*Sinecosyfis *sp.	*Chroomonas* sp.
1	YES	YES	YES
2	NO	NO	YES
3	YES	YES	YES
4	NO	NO	NO
5	YES	NO	YES
6	NO	NO	NO

**Table 5:** Kinetic growth behavior of microalgae presented in Figure 1.

Microalgae	Behavior at 20°C	Behavior at 32°C
*Dunaliella salina*	Linear, exponential, stationary	Adaptation, exponential, stationary
*Sinecosyfis *sp.	Linear, stationary	Linear, exponential, stationary
*Chroomonas* sp.	Linear, stationary	Linear, exponential, stationary

**Table 6:** Identified fatty acids and quantity of oil produced for the studied microalgae.

	Experiment number				
	1		2	3	
	Nutrifoliar medium, period of agitation and illumination for 24 hours, supplied 1620 L/day of air, temperature 32°C		Nutrifoliar medium, period of agitation and illumination of 12 : 12 hours, supplied 810 L/day of air, temperature 32°C	Nutrifoliar medium, period of agitation and illumination of 24 hours, supplied 1620 L/day of air, temperature 20°C	
Microalgae species	*Chroomonas *sp.	*Dunaliella salina*	*Chroomonas *sp.	*Chroomonas* sp.	*Sinecosyfis* sp.

Quantity of wet microalgae (g)	3	4	1.5	15.5	24

Oil produced (mg)	54.43	73.14	24.99	40.69	88.76

mg of oil/g of wet biomass	18.14	18.29	16.66	2.62	3.69

Extracted fatty acids	(1) Hexadecanoic acid, methyl ester. (palmitic acid) (2) 9,12-Octadecadienoic acid, methyl ester. Linoleic acid, omega-6 (3) Hexadecanoic acid, 1-(hydroxymethyl)-1,2-ethanediol ester. (4) 16-Octadecenoic acid, methyl ester (stearic acid).	(1) Hexadecanoic acid, methyl ester. (2) 4,7,10-Hexadecatrienoic acid methyl ester. Omega-6 (3) Octadecadienoic acid, methyl ester. (4) 9,12,15-Octadecatrienoic acid, methyl ester (Z, Z, Z). alpha-linolenic acid, omega-3 (5) 16-Octadecenoic acid, methyl ester.	(1) Hexadecanoic acid, methyl ester. (2) Hexadecanoic acid 1-(hydroxymethyl)-1,2-ethanediol ester. (3) Cis-11-eicosanoic acid, omega-9	(1) Hexadecanoic acid, methyl ester. (2) Octadecanoic acid, methyl ester. (3) 8,11-Octadecadienoic acid, methyl ester.	(1) Hexadecanoic acid, methyl ester. (2) Octadecanoic acid, methyl ester. (3) 8,11-Octadecadienoic acid, methyl ester
